# Management of embolic splenic abscess secondary to aortic valve endocarditis – case report and review of literature

**DOI:** 10.1186/s13019-024-02727-6

**Published:** 2024-04-16

**Authors:** Nicolas Nunez-Ordonez, Juan Sebastián Luna, Jaime Camacho Mackenzie, Andrés Felipe Jiménez, Alejandro González, Andrea J. Pico, Carlos F. Román, Paulo A. Cabrera Rivera, Carlos A. Villa Hincapié

**Affiliations:** 1 Cardiovascular Surgery Department, Fundación Cardioinfantil-LaCardio, Bogota, Colombia; 2 Cardiovascular Surgery Resident, Universidad del Rosario, Bogota, Colombia; 3 Universidad de la Sabana, Bogota, Colombia; 4 Chair, Cardiovascular Surgery Department, Fundacion Cardioinfantil-LaCardio, Bogota, Colombia; 5 Cardiovascular Surgery Fellow, Universidad del Rosario, Bogotá, Colombia; 6 General Surgeon, General surgery department, Fundacion Cardioinfantil-LaCardio, Bogota, Colombia; 7 General surgery resident, Universidad de la Sabana, Bogotá, Colombia; 8 Cardiovascular surgeon, Cardiovascular Surgery Department, Fundacion Cardioinfantil-LaCardio, Bogota, Colombia

**Keywords:** Endocarditis, Splenic abscess, Laparoscopic splenectomy.

## Abstract

**Background:**

Splenic abscess is a serious complication associated with infective endocarditis. There is still contradicting evidence regarding the optimal treatment pathway including timing of valve intervention and the approach for managing splenic foci.

**Case presentation:**

We present a case of a hybrid staged approach in which we successfully performed a laparoscopic splenectomy following percutaneous abscess drainage and a delayed aortic valve replacement.

**Conclusions:**

A multidisciplinary teamwork is fundamental in providing optimal care for patients with distant complications associated with infective endocarditis. Our hybrid approach seems safe and feasible.

## Background

Systemic embolism is a relatively frequent complication of infective endocarditis (IE). It has been estimated that 1 out of 4 patients with IE presents with some degree of systemic embolism, the most common sites being the central nervous system (CNS) and the spleen [1–3] However, splenic abscess formation is uncommon, presenting in up to 1% of all cases of IE [1].

The optimal treatment strategy for patients with aortic valve endocarditis with multiple systemic septic embolisms is still a matter of debate as balance between periprocedural risk and removal of septic foci must be obtained to ensure adequate outcomes [1]. Only a few cases or case series describing management of splenic abscess secondary to septic embolism have been published so far [4–14], with varying approaches and outcomes. We present a focused literature review based on a successful case of a multi-stage, hybrid approach for treating a patient with multiple systemic complications including a large splenic abscess secondary to native Aortic Valve Endocarditis.

## Case presentation

A 52-year-old male patient was referred to our institution with a diagnosis of a methicillin-susceptible Staphylococcus aureus native aortic valve endocarditis. He had a history of a recent posterior ischemic stroke with hemorrhagic transformation 2 weeks earlier with visual impairment, no motor deficit, and an initial Glasgow scale at 15/15. Upon admission he was in a fair general condition, tachycardic, febrile, without leukocytosis. A grade III diastolic murmur was detected.

**Fig. 1:**
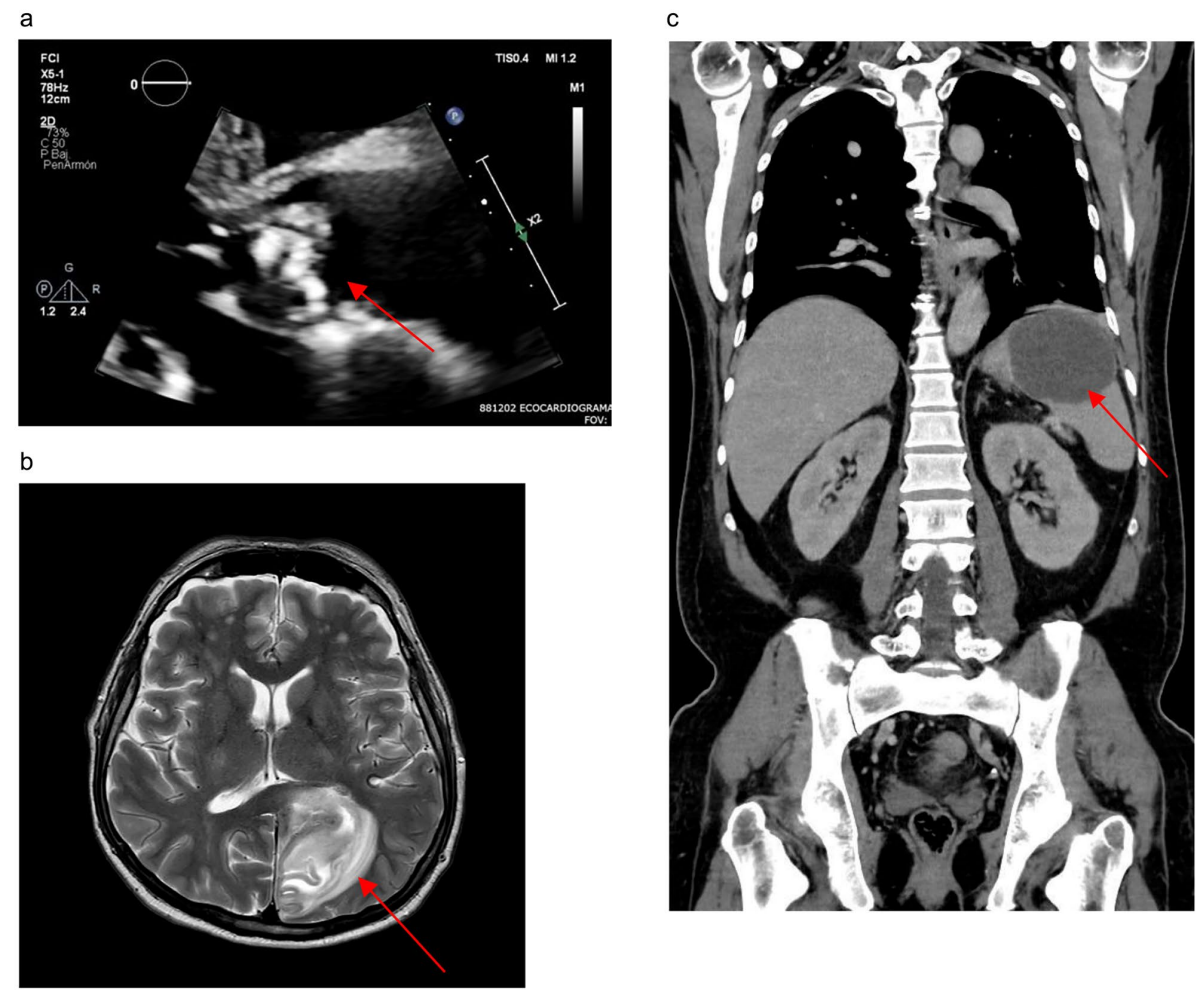
Imaging studies. (**a**) Transthoracic echocardiogram, showing a large vegetation of the aortic valve (red arrow). (**b**) Transverse view of brain MRI, showing late subacute infarction with small left occipital hemorrhagic transformation (red arrow). (**c**) Coronal view of abdominal CT, showing a large unilocular splenic abscess (red arrow)

Initial transthoracic echocardiogram showed a bicuspid aortic valve and the presence of a pedunculated vegetation which caused a severe aortic stenosis with moderate regurgitation. The left ventricle was mildly dilated with a preserved systolic function (Fig. 1a).

The patient was started on IV Daptomycin guided by blood cultures.

Due to valvular compromise, a surgical aortic valve replacement was considered, however the procedure was initially deferred given the evidence of recent CNS bleeding on admission MRI (Fig. 1b).

**Fig. 2:**
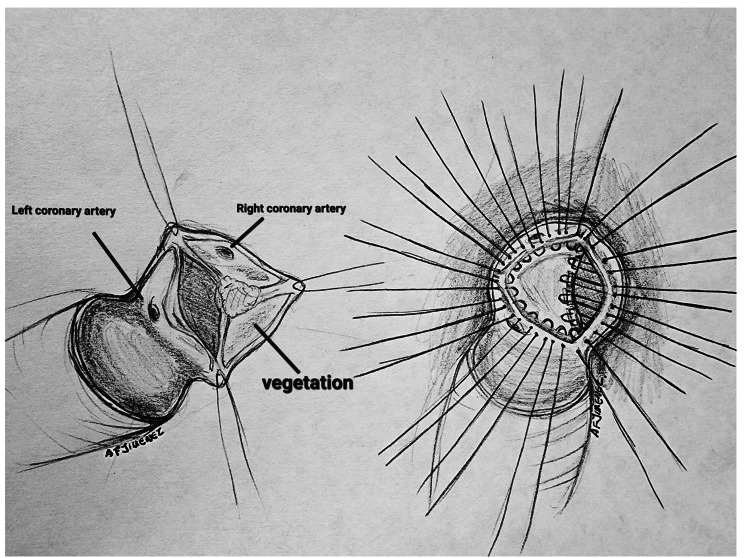
Schematic representation of intraoperative findings and procedure (*Source – Author AFJO*) (**a**) A calcified bicuspid valve was found with a large vegetation and an abscess extending through the aortic annulus. (**b**) Valve implantation technique. The abscess cavity was sealed with pledgeted sutures anchoring the prosthetic valve

Six days after admission, he presented with new onset fever despite receiving adequate targeted antibiotic treatment, new blood cultures and imaging studies were obtained. Brain angiogram showed no alterations. An abdominal CT revealed a large splenic abscess (Fig. 1c). After multidisciplinary consultation, valve surgery was further deferred aiming to achieve abdominal foci control. Given the radiological characteristics of the abscess, a high surgical risk was considered so a staged hybrid approach was decided. An initial percutaneous drainage of purulent fluid was performed aiming to reduce the size of the abscess and therefore reducing the chances of rupture and abdominal cavity contamination during a subsequent surgical intervention. The procedure was followed by a successful laparoscopic splenectomy three days later, finding an enlarged spleen with necrotic tissue and scarce purulent material. Cultures of the splenic abscess fluid were negative. After guaranteeing an adequate postoperative course, he was taken to a mechanical aortic valve replacement. Intraoperatively, a bicuspid aortic valve (fusion type with Noncoronary-right phenotype) was found with calcifications of the free edges and vegetations on the aortic side of the leaflets. An abscess of the aortic annulus which was extending from the left-right commissure towards the non-coronary-right commissure was found along with a small associated pseudo aneurysm at this level (Fig. 2a). After native valve removal and annulus debridement and decalcification, the abscess was drained. The annulus was sterilized with iodine-based solution and the small remaining cavity was sealed with the pledgeted sutures anchoring the prosthetic valve (Fig. 2b). He required a delayed sternal closure due to operative bleeding secondary to coagulopathy. During immediate postoperative care he required a pericardiocentesis due to cardiac tamponade and he received an implantation of a cardiac resynchronization therapy with a pacemaker (CRT-P) due postoperative grade III AV block. The rest of the postoperative course was uneventful. He was discharged with long term intravenous antibiotics. The patient has continued with strict follow-up, where he has shown no signs of relapse or complications after two-month follow-up. He received standard immunizations for post splenectomy patients which included pneumococcal and meningococcal vaccination along with seasonal influenza.

## Discussion

Systemic septic embolism is a relatively frequent complication of patients with infective endocarditis (IE), with an incidence ranging from 6 to up to 40% of cases [2, 3, 15], and it is a cause of significant morbidity and a mortality rate exceeding 20% [1, 6, 15, 16]. Some studies have reported an incidence of splenic abscess (SA) in this context around 1–8% [6, 10, 17]. Vegetations larger than 20 mm and a preoperative white blood cell count > 12.000 have been identified as risk factors for splenic infarction [1]. According to some studies, less than 5% of these cases will develop a SA, mainly associated with persistent bacteremia [1, 6, 17].

Persistent fever or bacteremia despite adequate antimicrobial therapy has been identified as one of the most common clinical manifestations of SA as other symptoms are nonspecific and infrequent [1, 6]. Therefore, an adequate, timely diagnosis requires a low threshold for imaging screening of septic complications in patients under management of IE. In our case a contrast-enhanced abdominal CT provided useful information allowing targeted decision-making. It has been shown that its sensitivity and specificity range around 90–95% for distinguishing splenic infarction from SA [6, 11, 17] and it has been proposed as the ideal screening study for patients with the previously mentioned high-risk features [15]. Ultrasound, MRI, or PET have also been reported in literature as an adjuvant for adequate diagnosis and surgical planning [1, 13, 18, 19].

Guidelines for treating patients with SA secondary to IE don’t specifically address the optimal management that these patients should receive as there is insufficient high-quality evidence [1]. According to available literature and from our experience, the main factors that must be considered include:

1. Timing of Aortic Valve surgery as compared to splenic interventions.
2. Choosing an adequate approach for controlling the splenic inoculum.
3. Choosing optimal medical therapy.

**Fig. 3:**
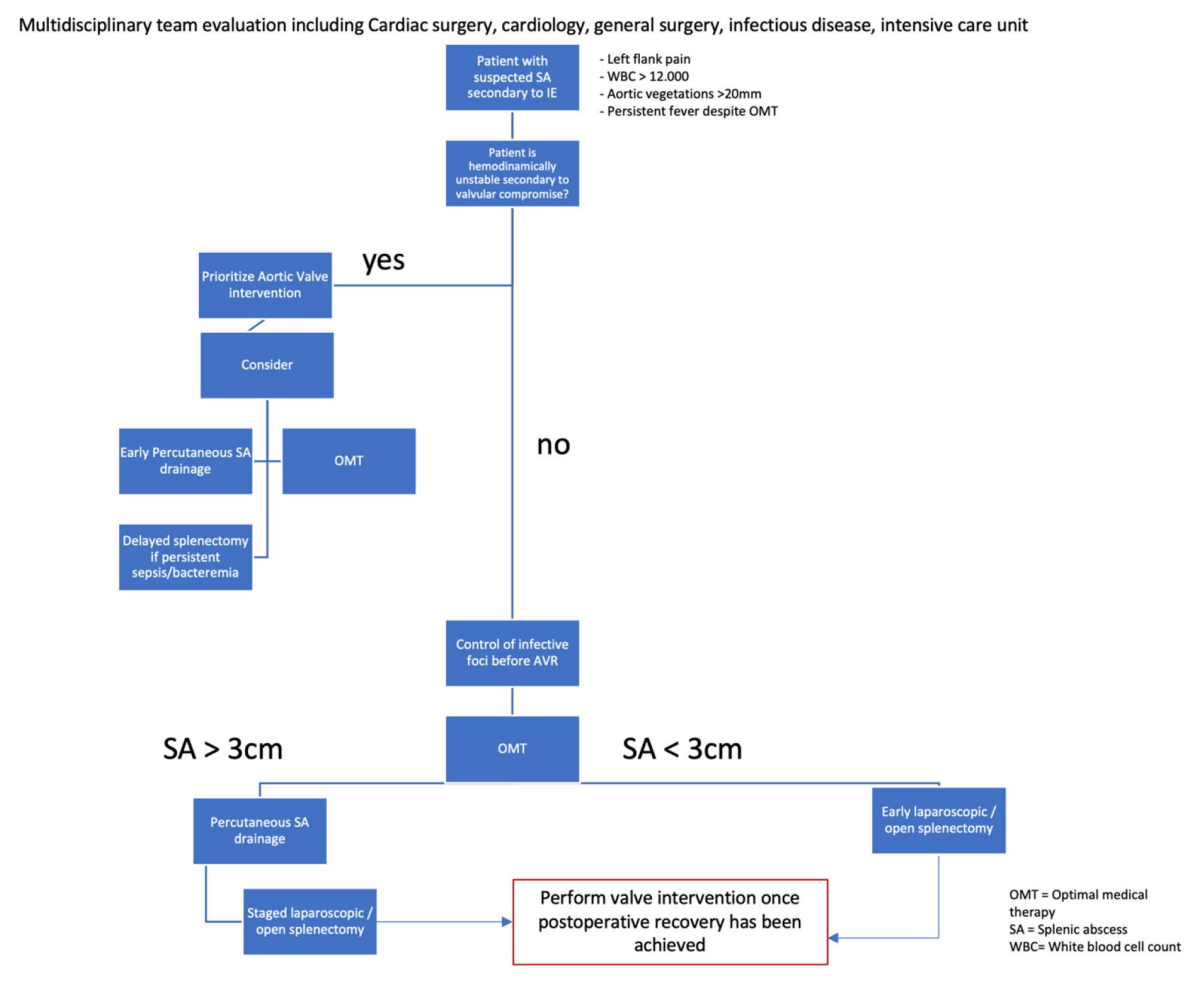
Proposed Management pathway for patients with splenic abscess secondary to infective endocarditis

We provide a proposal of a management pathway for patients with IE and SA (Fig. 3). Several approaches have been proposed regarding timing of valvular and splenic intervention [5–8, 12, 16, 20–23]. Table 1 provides a focused summary on the different management pathways that have been used in reports so far.

**Table 1:** Focused review of published cases of splenic abscess in bacterial infective endocarditis

Study	Number of patients	Management	Outcome
Conservative management (medical therapy only)			
Jolobe OMP et al., 1983 [22]	1	Antibiotics	8 week CT follow-up, complete resolution of splenic abscess
Wang CC et al., 2009 [9]	1	Antibiotics	No mention of valve intervention 1 year follow-up, no complications
Park S et al., 2009 [24]	1	Medical Therapy only	No recurrence
Alnasser SA et al., 2019 [6]	1	Antibiotics Emergency valve replacement	8 week follow-up CT showed smaller splenic abscess 1 year CT showed complete resolution 3 year follow-up no recurrence.
Nallarajah J et al., 2020 [25]	1	Antibiotics	Prolonged hospital stay No relapse at 2 years
Percutaneous drainage, no splenectomy			
McOwat L et al., 2015 [19]	1	Antibiotics Percutaneous drainage.	Unsuitable for splenectomy due to comorbidities No valve intervention. Biochemical marker improvement before discharge.
Ulloa N et al., 2020 [10]	1	Antibiotics Percutaneous drainage Valve replacement	Discharged without complications
Saijo F et al., 2022 [8]	1	Antibiotics Percutaneous drainage Valve replacement	13 month follow-up, no complications
Tsurui T et al., 2022 [21]	1	Antibiotics Percutaneous drainage	No information on valvular interventions 4 month follow-up CT showed splenic abscess reduction in size.
Required surgical intervention on the spleen			
Yoshikai M et al., 2002 (case 1) [16]	1/2	Antibiotics Emergent valve replacement Open splenectomy same surgical time.	4 year follow up No complications
Yoshikai M et al., 2002 (case 2) [16]	2/2	Antibiotics Valve replacement Open splenectomy same surgical time	1 year follow up No complications
Yilmaz MB et al., 2003 [26]	1	Antibiotics Laparoscopic splenectomy Valve replacement	No postoperative complications
Simsir SA et al., 2003 [20]	1/2	Antibiotics Laparoscopic splenectomy Valve replacement	1 year + 9 months follow-up, no complications.
Simsir SA et al., 2003 [20]	2/2	Antibiotics Percutaneous drainage Laparoscopic splenectomy Valve replacement	2 years + 4 months follow-up. No complications.
McCready RA et al., 2007 [27]	1	Antibiotics Valve replacement Open splenectomy (Same surgical time)	17 month follow-up without recurrent infection
Naito R et al., 2010 [17]	1	Antibiotics Open splenectomy Valve replacement	6 month follow-up, no complictions
Elasfar A et al., 2015 (case 1) [13]	1/3	Antibiotics Splenectomy Valve replacement	9 month follow-up, no complications
Elasfar A et al., 2015 (case 2) [13]	2/3	Antibiotics Valve replacement Emergency open splenectomy	8 month follow-up, no complications
Elasfar A et al., 2015 (case 3) [13]	3/3	Antibiotics Splenectomy Valve replacement	5 week follow-up, no complications
Blasi S et al., 2016 [11] (case 1)	1/3	Antibiotics Percutaneous drainage Laparoscopic splenectomy Valve replacement	2 year + 9 month asymptomatic
Blasi S et al., 2016 [11] (case 2)	2/3	Antibiotics Laparoscopic splenectomy Valve replacement	8 month asymptomatic
Blasi S et al., 2016 (case 3) [11]	3/3	Antibiotics Open splenectomy Valve replacement (Same time)	1 month asymptomatic
Aalaei-Andabili SH et al., 2017 [15]	33 (17 splenic only + 16 splenic and CNS embolism)	11 splenectomies 5 before cardiac surgery 4 concomitant 2 after. 22 antibiotics + CT follow-up	8,7% overall mortality Higher AKI in splenectomy group. No additional interventions required after discharge for medical therapy only group.
Lindsey ME et al., 2017 [28]	1	Antibiotics Percutaneous drainage Emergent Valve replacement Robotic assisted splenectomy.	Splenectomy performed 12 weeks after IV treatment due to persistent splenic collection to prevent re-infection. 2 year follow up no complications.
Groga-Bada P et al., 2018 [18]	1	Antibiotics Splenectomy	No valve intervention No information on follow-up.

### Timing of aortic valve surgery

There is still controversy around the optimal timing of valve surgery and high-quality studies are lacking [14]. Two factors must be balanced to make adequate clinical decisions: the risk of hemodynamic compromise secondary to valvular damage on one hand, and the risk of prosthetic valve infection if a valvular replacement is undertaken while the patient has active septic foci despite optimal medical therapy (OMT) [6, 16].

Double intervention targeting both the infected valve and the spleen has been reported and it is not a new approach [16, 27] however it is most commonly believed that this operation may represent a significant physiological stress that may put patients at risk [6, 15, 17] so staging the interventions, performing valve surgery after resolution of abdominal foci has been obtained is often preferred [4, 11, 13, 16, 28] although there is contradicting evidence around this topic [7, 8].

This case had a recent embolic stroke with hemorrhagic transformation as a particular complication for deciding the optimal timing for cardiac surgery. Current guidelines recommend deferring surgery for > 1 month after hemorrhagic central nervous system (CNS) events (class IIa) however surgery can be prioritized if a persistent infection, high embolic risk or hemodynamic instability are determined (class IIa) [1]. Surgery was decided after neurology and neurosurgery consultation and CNS imaging. AVR was performed at least 1 month after the detection of the hemorrhagic transformation which occurred before referral to our institution.

### Choosing an adequate approach for controlling the splenic inoculum

Complete treatment for SA should be actively seeked as this reduces the risk of valvular prosthesis infection. Performing a splenectomy is not mandatory. Some reports have shown satisfactory results with a conservative treatment consisting of a long intravenous + oral antibiotic regimen [7, 10, 23–26] or in combination with percutaneous drainage [21]; however, the multidisciplinary group at our institution decided to minimize the risk of complications after aortic valve intervention by performing a full splenectomy as it has been associated with higher survival rates [4, 11, 17, 29, 30].

Percutaneous drainage is a safe approach with minimal morbidity [8, 21]. No clear indications for this procedure have been established so far, however it should be considered in cases of solitary, unilocular SA larger than 3 cm [21].

Both the laparoscopic and open splenectomy have proven effective in treating this group of patients [20, 23, 28]. Our report provides insight on a hybrid, multi-stage approach in which, due to the volume of the SA, we decided to perform a preoperative percutaneous drainage of the SA to minimize blood loss, risk of cavity contamination, SA rupture and overall surgical risk while improving the chance of a successful laparoscopic splenectomy [28] for definitive removal of the infection. In this setting, a minimally invasive laparoscopic approach for the SA has the advantage of allowing a faster recovery for a subsequent valvular intervention [12, 23].

Performing safe laparoscopic surgery in the setting of severe aortic stenosis might be challenging and therefore it is suggested that the anesthesiologists are familiar with intraoperative management of this group of patients.

### Choosing optimal medical therapy

This is the cornerstone for IE management. Specific guidelines on choosing and adequate medical therapy have recently been published and are beyond the scope of this manuscript [1, 4, 26]. A management algorithm has been recently published by the ESC regarding medical therapy according to the microbiological profile and choosing an adequate timing of surgery accordingly [1].

## Conclusions

Treating patients with splenic septic embolism in the setting of IE is still a clinical and surgical challenge. A wide range of interventions is available and should be chosen on a case-by-case basis. This requires articulation of a multidisciplinary team integrating adequate medical therapy, a timely splenic intervention and delaying aortic valve interventions as much as possible according the hemodynamic and inflammatory state of the patient while allowing adequate control in extracardiac infective foci. Our multi-stage hybrid approach involving optimal antimicrobial therapy, percutaneous drainage, a laparoscopic splenectomy and a delayed AVR guaranteeing an improved clinical condition seems to be feasible and safe.

The current report is aimed at helping guide clinical decision-making by providing a framework for choosing an appropriate treatment pathway. Given the difficulty of implementing large randomized clinical trials in this kind of settings, we believe that clinicians should aim at standardizing and recording multidisciplinary institutional protocols for complex IE cases presenting with systemic embolic complications. Timely report of experiences can improve patient outcomes by providing a strong body of evidence allowing higher-quality information to be developed.

## Data Availability

No datasets were generated or analysed during the current study.

## Abbreviations

AVR: Aortic valve replacement
CT: Computed tomography
CRT: P-cardiac resynchronization therapy with a pacemaker
IE: Infective endocarditis
MRI: Magnetic resonance imaging
PET: Positron emission tomography
OMT: Optimal medical therapy
SA: Splenic abscess
